# Marine Polysaccharides in Microencapsulation and Application to Aquaculture: “From Sea to Sea”

**DOI:** 10.3390/md9122572

**Published:** 2011-12-08

**Authors:** Massimiliano Borgogna, Barbara Bellich, Attilio Cesàro

**Affiliations:** Department of Life Sciences, University of Trieste, Via L. Giorgieri, 1-I-34127 Trieste, Italy; Email: mborgogna@units.it (M.B.); bbellich@units.it (B.B.)

**Keywords:** polysaccharide properties, conformation and dynamics, hydrogels, encapsulation, fish vaccination

## Abstract

This review’s main objective is to discuss some physico-chemical features of polysaccharides as intrinsic determinants for the supramolecular structures that can efficiently provide encapsulation of drugs and other biological entities. Thus, the general characteristics of some basic polysaccharides are outlined in terms of their conformational, dynamic and thermodynamic properties. The analysis of some polysaccharide gelling properties is also provided, including the peculiarity of the charged polysaccharides. Then, the way the basic physical chemistry of polymer self-assembly is made in practice through the laboratory methods is highlighted. A description of the several literature procedures used to influence molecular interactions into the macroscopic goal of the encapsulation is given with an attempt at classification. Finally, a practical case study of specific interest, the use of marine polysaccharide matrices for encapsulation of vaccines in aquaculture, is reported.

## 1. Introduction

The polymer science viewpoint has been extended in the last two decades to many other fields of research, from food science and technology to pharmaceutical and biomedicine application. 

It is a first principle in the general polymer textbook that, when specific interactions are weak or absent, most polymers are by definition incompatible and show a tendency to undergo liquid-liquid phase separation. In addition to the phase behavior enhanced either by a decrease or an increase in temperature, some polymers may also have further elements of topological complexity in their ordering processes which make the event of phase separation more complex. Thus, understanding solution phase behavior and associative phenomena of mixed polymers, and biopolymers in particular, is important from many viewpoints. The thermodynamic phase boundary location and the dynamic aspects of phase separation are related to the non-equilibrium relaxation processes that follow the perturbation of the system from a thermodynamically stable to a thermodynamically unstable state. Indeed, these processes are usually induced not only by changes in temperature, but also solvent (concentration or composition) or pressure. Polymer compatibility, in the sense of thermodynamic stability, depends in a subtle way on molecular parameters, both intrinsically conformational and energetic. As a general principle, two high molecular weight polymers are mutually incompatible in the absence of favorable interaction. Of a great practical interest is the situation when one or both polymers are weakly charged and modulation of the solution compatibility can be achieved by tuning the ionic strength or pH [1]. Some aspects of these phenomena are briefly described in the following sections, reporting in particular on the phenomenon of polymer complexation and gelation of weakly charged polysaccharides and their self-assembly in forms of beads (from nano to micro) for several practical applications, including the delivery of vaccines in aquaculture.

The intent of this review is to examine a progressive field of research through the authors experience and interests; therefore it is not necessarily exhaustive or comprehensive. The paper is organized in part in a semi-tutorial way, from the description of some basic blocks of polysaccharides (glycans) to form chain topologies and supramolecular structures that can usefully provide encapsulation of drugs, for application in vaccines. Thus, the following section reports on several molecular aspects involved in the construction of polymeric self-assembled structures, aiming at the basic phenomena behind the bench work. Then, by introducing the requirement of biocompatibility and, moreover, of biodegradation and safety of the polymeric matrices, an analysis of some polymeric categories generally regarded as safe (or GRAS) is provided, illustrating in particular those polymers that are largely used, both from the historical and from the economical viewpoint. The third section illustrates the way the basic physical chemistry of polymer self-assembly is made in practice through laboratory methods, by coarsening the fine tuning of the molecular interactions into the macroscopic goal of encapsulation of a macromolecular drug. Finally, the last section is devoted to a case study of specific interest, the use of polysaccharidic matrix for encapsulation of vaccines for aquaculture, by exploring the formulation and the characterization of several systems.

## 2. Polysaccharides

### 2.1. Structure and Shape Determinants for Aggregation and Gelation

This work points out the biophysical rules in the construction of nanostructures with application in food, cosmeceutical and pharmaceutical fields. Attention is mainly given to the polysaccharide conformational and solution properties which are at the basis of the self-interaction processes generating nano- and micro-structures. The main reason for focusing attention on polysaccharides is certainly due to their versatility accompanied by the fully biocompatibility and biodegradability. A description of the topological and dynamical parameters characterizing the conformational properties of polysaccharides has been presented elsewhere [2] and is summarized in Section 2.3. The relevance of spatial and temporal concepts has been clearly pointed out in a recent paper dealing with the statistical conformational analysis of glycans. This problem was summarized in the question “What conformational states does the glycan adopt and what are their population?” as opposed to the other simpler one “What is the shape of my glycan?” [3].

The solution properties of biopolymers, and polysaccharides in particular, have always been of considerable interest for many commercial applications, such as thickening, suspending and gelling agents [4,5]. The wide range of rheological behavior shown by polysaccharides in aqueous solution is due to the variety of conformations and chain flexibility, spanning from the more or less expanded coil (like dextran or guar) to the rigid rod limit (like xanthan) which is peculiar for this class of polymers. Still lacking is, however, the direct correlation of the chemical structure and the conformational features of the chains with the rheological behavior displayed, although experimental and simulation studies have been addressed more recently to understanding the process of elasticity and dissipation at nanoscale level by means of atomic force microscopy and conformational analysis [6,7]. The main reason to refer to rheological properties is due to the clear definition that the ratio of the elastic modulus to the dissipative viscous one gives for the identification of the presence of a gel phase, often called “soft condensed matter”. A gel phase occurs as an irreversible phenomenon for chemically cross-linked polymer chains, while it is a consequence of solvent or temperature perturbation in many other cases, classified as “reversible gels”. 

The formation of thermoreversible (physical) gels has been observed in many dilute polymer solutions and has previously provoked an enormous interest because of both the fundamental aspects of the morphology and of the gelation kinetics of these systems [8,9,10]. Polymer physical gels are also formed by crystallization from quiescent solutions, while for a time the belief persisted that, apart from the biopolymer gel networks [8], shearing was required. As a notable example, the crystals formed during gelation of many synthetic polymers are of lamella type, like those formed from dilute solution [11]. 

According to experimental findings in gel formation, many variables are involved to control the resulting structures, in particular, cooling rate, gelation temperature, and solvent properties, which are all important for the crystallization process. As the solution is cooled, chains can form crystalline domains that serve as reversible cross-links if the chain entanglement in the solution is sufficiently developed. This scheme is in line with Flory’s concept of the minimum functionality which can generate cross-linking gelation [12] and with the statistical model of Tanaka and Stochmayer [13]. 

**Figure 1:**
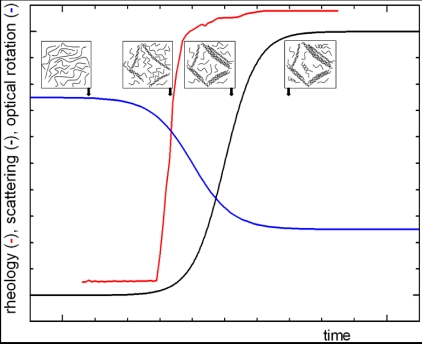
General mechanism (nucleation and growth) of gel formation as macroscopically measured by viscoelastic, optical and chiroptical properties. The insets show sketch of the polymer solution microstructure at the various stages.

Recent results reported in the literature [14,15,16] provide the basis for a better understanding of the gel structure and gelation mechanism for many systems, either synthetic or natural polymers. The mechanism of thermoreversible gelation of polysaccharides in solution is investigated through the study of the temperature-concentration relations, the thermodynamic behavior of the solvent and the gel melting, the change in molecular conformation by several spectroscopic methods and structural and dynamic analysis by light scattering. In particular, the time dependence of structural and dynamic parameters can be measured after a rapid quenching of the polysaccharide solution at a given concentration (above the critical threshold), providing a picture such as schematically reported in Figure 1. The mechanism consists of different steps: a coil to (double) helix transition followed by an intermolecular association that finally leads to a macroscopic network like a crystal lattice. 

Models for fibrillar aggregation arise especially from the polysaccharide field and have also been pictured by several microscopy methods in the case of gellan, agarose, carrageenan, and other relatively stiff chains [14,17,18]. Many of these biopolymers have low flexibility (e.g., worm-like polysaccharides with persistence length Lp > 60 nm) and show a remarkable deviation from the solution behavior described for flexible molecules. They often undergo gelation in a cooperative process triggered by small changes in temperature, ionic strength, pH [8,19]. A similar suggestion has also been put forward for polystyrene gels [20], in which the complexation with the solvent is the crucial factor affecting the chain stiffening. However, neither the solution features nor the gel morphology and behavior exhibited by many biopolymers can support the molecular mechanism or the conformational motifs as those ascribed to stiff polysaccharides and solvent-complexed polystyrene. 

### 2.2. Thermodynamic Considerations

As chain dimension and stiffness not only depend on structural characteristics but also on the solvent quality, the other point to discuss is the more general thermodynamic aspect of the gel. In physical gelation processes, the important driving force is the conformational ordering of segments of chains which reach a critical size for the Gibbs free energy stability. The dimensionality of the gel nucleus is again governed by the stiffness of the chain. On this basis, it does not seem appropriate to assume that very flexible chains which are known to form gels in dilute solutions are organized with the same mechanism proposed for rigid polymers. Therefore, both in biopolymers and synthetic polymers the process of local crystallization and gelation can occur either in the lamella-like (e.g., starches) or fibrillar-like (e.g., gelatin) organization. 

**Figure 2:**
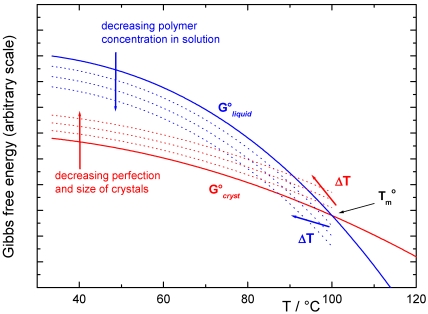
Schematic representation of the Gibbs free-energy curve *vs*. temperature for the liquid phase (as a function of composition, blue lines); and for the crystalline phase as a function of decreasing crystal size (red lines). The practical effect is a decrease in the measured melting temperature.

Independent of the structure of crystalline junctions, the size of the ordered gel phase is a relevant aspect which determines the thermodynamic stability and therefore the changes in the melting temperature Tm of the gel phase. The other is the presence of a component (solvent or plasticizer) which acts as a solvent for the amorphous/liquid phase in equilibrium at Tm with the solid crystalline phase. The first development of a theoretical formulation was given by Flory and Mandelkern [21] and uses the Flory-Huggins model for the thermodynamic properties of the polymer solution. However, this is not sufficient for many cases, where the crystalline phase is formed by crystals whose dimension and morphology are dependent upon the experimental conditions [22]. In a schematic representation of the Gibbs free-energy diagram *vs*. temperature, the energy surface is described by a family of curves for the liquid phase (*i.e.*, the Gibbs free-energy curve of the liquid polymer and those of the pure liquid polymer with increasing concentration of the other component) and also by a family of curves for the solid phase (*i.e.*, the Gibbs free-energy curve of the perfect crystal of infinite size and those of the crystals with an increasing number of defects and decreasing size). Melting temperatures, such as those in Figure 2, are then a function of both the composition of the system and the dimensional features of the crystal (*i.e.* by the Gibbs-Thomson-Tamman equation), as recently “rediscovered” in the field of nanomaterials. Many literature results confirm this general interpretation, although they are not always reported in this form. 

Having discussed how the overall process of chain-chain interaction is governed by chemical and physical parameters, it is also worth pointing out the principles that shape the polymer aggregates for specific functions. Indeed, the most important feature for spherical gel particles is not only the diameter (from millimeters to nanometers) but also the distribution of polymer aggregates as the actual texture may change from the interior to the surface. To make this clear with one single example, it is sufficient to mention the relevance of survival of a cell encapsulated within a gel particle in terms of dimension of the confined cavity and of the extent of diffusion of metabolites and catabolytes, in addition to the proper environmental chemical properties. For the above reasons, gel systems in the biological fields (especially for applications in foods and pharmaceutics) are mainly based on biopolymers and polysaccharides in particular. 

Finally, in several less stringent applications, other kinds of gels are prepared from synthesis of natural polymers through chemical cross-linkers or by the free radical polymerization of monomers in the presence of a bifunctional cross-linking agent. New classes of gels with two levels of structural hierarchy have also been engineered by first making gel nanoparticles and then covalently bonding them together. In this way the primary network is made by cross-linked polymer chains in each individual particle, while the secondary network is a system of cross-linked nanoparticles. Such nanostructured gels have appealing properties that conventional gels do not have, including a high surface area, a bright color at room temperature, and temperature-tunable heterogeneity on the nanometer scale [23].

### 2.3. How Do Polysaccharide Chain Solution Properties Depend on Sugar Monomer Composition?

**Figure 3:**
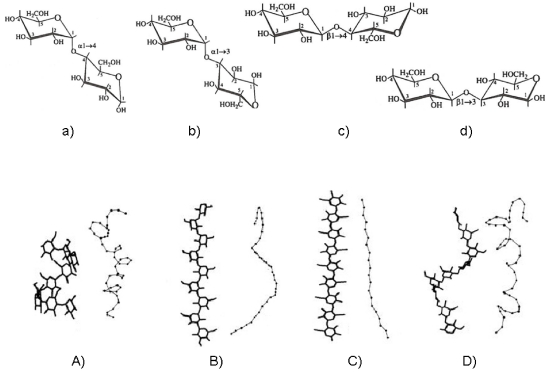
Structural representation of four disaccharides from glucose: (**a**) maltose (α-(1-4)-linked); (**b**) nigerose (α-(1-3)-linked); (**c**) cellobiose (β-(1-4)-linked); (**d**) laminarabiose (β-(1-3)-linked). The related polysaccharide structures of amylose (**A**), nigeran (**B**), cellulose (**C**) and laminaran (**D**) are shown in the ordered helical conformations as measured by X-ray fiber diffraction studies (left) and as snapshots of random chains modeled by Monte Carlo calculation (right).

It is now well known that linear glycans, differing from one another only in glycosidic linkage position and stereochemistry, can display a wide range of equilibrium conformational features [24,25]. The variability of chain flexibility arises from the large number of naturally available sugar residues with several linkage patterns and from the possible occurrence of intra- and intermolecular interactions. The former controls the overall chain geometry and the latter governs the pattern of shapes and dimensions characterizing the polymer in solution. Under given conditions the above constraints may induce regular helical conformations to some extent. A “portfolio” sketch of how the main structural blocks of polysaccharides prefigure the local conformational features and therefore the chain topology is given in Figure 3. 

Commonly, some of these well known equilibrium conformational differences are schematically illustrated by the typical stiff and extended (1→4)-β-linked chain of cellulose [26,27] and similar “cellulosic” chains, like chitosan [28]. Other features are exhibited by the “pseudo-helical” (1→4)-α-linked random coil chain of amylose [29] or by the more disordered open helical-like that of the (1→3)-β-linked chain of curdlan [25,30]. Additional examples of chain topology are provided by industrially important polysaccharides, like hyaluronan [31,32], schizophyllan [6] and scleroglucan [7], which have been modeled with a reasonable level of accuracy.

Although schematically simple in the statements given above, it is more complicated in practice to experimentally develop structural (molecular) understanding of the conformational mobility and dynamics of oligo- and polysaccharides in solution. While modern Molecular Dynamics investigations provide a huge amount of numerical simulations, the underlying physical rules are hidden behind the “beauty” of the molecular figures the computation productions. 

For an implementation of these pictorial views, the time dependence of the conformational changes in solution has to be considered as this is likely also to be dependent on the features of the glycosidic linkages [7,31,33]. The full physical description of all these local motions are conveniently given by the spectral density of ^13^C–^1^H bond motions in the nanosecond frequency range [34,35,36]. Thus, some correlations between the conformational dynamics and the structural details have been sought, although systematic theoretical investigation of the structure dynamics relationships for naturally occurring sugar sequences is still in progress [37].

In an attempt to scrutinize the above features, the very preliminaries for a correct setting of knowledge about the shape of polysaccharides in solution are essentially given by the correlation between three contributions: the primary structure (*i.e.*, the chemical identity of the carbohydrates polymerized in the chain), the intrinsic conformational features dictated by the rotational equilibrium (often the major contributions are due to the rotation about the glycosidic linkages) and the interaction with the other molecular species in the system, *i.e.*, solvent and cosolutes [38]. 

As already mentioned, the primary structure of polysaccharides is complicated by different kinds of linkages in homopolymers and different kinds of monomer units, facts which give rise to a huge number of different polymers. Glucans (see a general formula in Figure 3) are those composed exclusively of glucose, while glucuronans are polymers of glucuronic acid. Similarly mannans and galactans as well as mannuronans and galacturonans, are homopolymers of mannose, galactose, mannuronic acid and galacturonic acid, respectively. Although the listed polysaccharides have a structural regularity (homopolymers), many other glycans are not homopolymers and their chemical structure can often be fairly complicated, as in the case of microbial and plant polysaccharides [39,40,41].

From the experimental point of view, several omo- and co-polysaccharides with different chain linkage and anomeric configurations have been studied to determine to what extent the polymeric linkage structure and the nature of the monomeric unit are responsible for the preferred solvation and for the chain topology and dimensions [42]. Conversely, since it is generally understood that the structure and topology of many macromolecules are affected by solvation, theoretical models must include these solvent effects in addition to the internal flexibility, in order to estimate changes in the accessible conformations as a result of the presence of the solvent molecules. The practical problem is to what extent do solvents and co-solutes contribute to stabilizing some conformational states rather than others. Since solution properties reflect the distribution of conformations of the chain molecules, the experimental data are the unique solution to the statistical thermodynamic average over all the accessible conformational states of the molecule. This aspect has been illustrated by taking into consideration the accessible conformational states of a simple sugar unit “*in vacuo*” and the conformational perturbation arising from a solvent medium [43,44,45,46,47,48,49,50]. Therefore, in most realistic situations, account must be taken of the conformational perturbations given by specific molecular interactions. These interactions often modulate the conformation to a local minimum and impose some structural constraints giving long range pseudo-order. The naïve response to these questions, which requires a complete knowledge of the time-space dependence of the chain topology, is often “rounded-off” by the use of empirical terms like “flexibility”. 

Passing from monomer units to oligomers (disaccharides and higher oligosaccharides), the dominant features of molecular flexibility become those due to rotations about the glycosidic linkages. Although all other conformational fluctuations contribute to the local dynamics of the atoms or group of atoms, only the glycosidic linkage rotations are able to dramatically change the conformational topology of oligomers at ambient temperature. The aim of a conformational analysis of oligosaccharides is thus to evaluate the probability (that is the energy) of all mutual orientations of the two monosaccharide units, as a function of rotations about the glycosidic linkages, defined by the dihedral angles φ and ψ. The important regions of φ and ψ rotations are those with energy variations in the order of kT, the thermal motion energy, because this may produce a large ensemble of accessible conformational states for the oligosaccharide. There are some contrasting interpretations as to whether the presence of water contributes to the increase or decrease of the glycosidic conformational freedom. This is sometimes due to the possibility of contribution of water molecules bridging two adjacent sugar rings, an event which is more probable in stiff disaccharides than in the more flexible 1→6 linked disaccharides. 

Even when the rotational motion is restricted to only a few angles, the fluctuations of many such glycosidic bonds is amplified along the chain backbone, as the molecular weight increases. The accumulation of even limited local rotations may produce very large topological variations in the case of polymeric chains and consequently relevant changes in thermodynamic, hydrodynamic and rheological properties of these systems. Other internal motions often make only small contributions to the observable properties on the macromolecular scale [26]. Attempts to take into account the full range of conformations have been made in the past in order to predict some suitable set of experimental data, like hydrodynamic volume and radius of gyration, and, more recently, Small-Angle X-ray Scattering results [47,51]. 

### 2.4. Solutions of Ionic Polysaccharides

The solution behavior of ionic macromolecules (polyelectrolytes) is by far more complicated. Based on the experimental evidence of polyelectrolyte solutions, all high molecular weight ionic macromolecules are characterized by a peculiar behavior which sets them apart from all other ionic low molecular weight molecules as well as from non-ionic macromolecules. A general consequence of the presence of charged groups on the chain is a favorable contribution to the solubility of polymer in water. Also, a strongly attractive potential is generated between the charge density on the polymer and the opposite charges in solution. As a consequence, the value of the activity coefficient of the counterions is strongly reduced with respect to that of the same ions in the presence of the univalent opposite charged species. When the ionizable groups are rather weak (e.g., carboxylic groups), the behavior of the polymer is a function of pH (*i.e.*, of the degree of ionization α). For these reasons, the study of the protonation-deprotonation process of a carboxylic polymer is an efficient tool for understanding the various contributions and for revealing, through the change of charge density, variations occurring in the intramolecular and intermolecular interactions. However, because the flexibility of conformation alters the distances between charged groups on the polymeric chain, a new equilibrium is statistically defined by the total Gibbs energy minimum of the system [52]. As an important consequence of this energy balance, changes in temperature, ionic strength, pH, *etc*., can provoke changes in polyelectrolyte conformation and dimension, often cooperatively in many biopolymers, between states with different values of the charge density. These states may be characterized by different structural orders (e.g., helix→extended chain transition), by different degrees of flexibility of the chain (globular coil→expanded chain) or by different extents of aggregation (monomeric→dimeric or multimeric chains).

For the interpretation of the experimental data and for the understanding of the correlation between properties and structure, a theoretical approach based on a molecular model is necessary. The central problem is that of quantifying the interactions among charges on the polymer and among these and their respective counterions. The most common approaches are those which describe the linear polyelectrolyte using a model with local cylindrical geometry. Although no derivation is given here of the thermodynamic functions associated with the process of “ion binding” [53,54], let us briefly state that a polyelectrolyte approach, based on the counterion condensation theory developed for dilute solutions by Manning [55], predicts a positive enthalpy change on mixing the polymer with a simple salt. Under the same circumstances, a positive volume change and a negative entropy change are also predicted for the merely electrostatic interaction of the point charges with the linear polyelectrolyte. 

**Figure 4:**
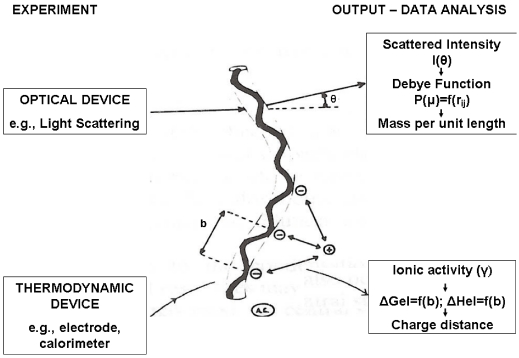
Correlation between structural conformational data obtained by scattering experiments (top) and structural data obtained by molecular thermodynamic theories (bottom). The example refers to the chain mass-per-unit length (from light scattering) and to the average distance between charged groups (from potentiometry or calorimetry).

The measured enthalpy of binding evaluated by direct calorimetric experiments has been published for different cations and polysaccharides and nicely confirms the predicted behavior, when other conformational changes or aggregative processes are absent. In other cases, scrutiny of the dependence of the experimental enthalpy changes as a function of the degree of complexation (or of the ion-to-polymer molar ratio) discloses cooperativity (binding and/or state transition), that easily monitor the occurrence of a chain conformational transition [56], in particular if the dependence is contrasted to the theoretical trend of the purely polyelectrolytic binding. Furthermore, the analysis can detect the actual charge density of the ionic polysaccharides (either single chains or multiple chain aggregates), since the free mono- or di-valent cation effectively acts as a “thermodynamic probe” of the true chemical potential associated to the structural characteristics of the charged species. Figure 4 illustrates this concept of the ion as a thermodynamic probe by comparing the structural information obtained by scattering experiments with that achievable by molecular thermodynamic experiments, both using suitable theories. 

Within this perspective, the theoretical derivation of the Gibbs energy, enthalpy and volume change on mixing a polyelectrolyte with monovalent and divalent ions has been explored for many cases, e.g., for carrageenans with Cs^+^ [54,57], always compared with experimentally measured values of the same properties. It is also clear that the volume change, as experimentally observed, may be larger than that which would be calculated on the basis of theory alone, since no provision is made in the theory for the volume change upon ion desolvation. 

As far as it concerns the short-range interactions in the absence of aggregates or gel formation, the introduction of charged groups modifies the equilibrium geometry of the monomeric units and the contribution of the electrostatic nature on the nearest-neighbor conformational energy. There are at least two approaches that are relevant for this review; one is that described by Haug and Smidsrød [58] for the rationalization of the dimensional properties of polyelectrolytes as a function of salt concentration, the other is the already mentioned formulation of a statistical thermodynamic theory for the “physical” framing of the ion-polyelectrolyte interactions. Both these theoretical formulations deal with the conformation of the polymer and predict that the conformational features must be a function of ionic strength (see for example [53,59]). More recent developments in the exploitations of structure-property-applications of ionic polysaccharides [60,61,62,63] make indirect use of the above concepts.

### 2.5. Marine Polysaccharides: Alginate and Chitosan Solutions

Some aspects of the main phenomena that describe the conformational properties, and their perturbation, of carbohydrate polymers have been covered in this section. One of the major concerns has been to describe these “effects” starting with the complex conformational equilibria generated in the simple chains. In fact, only recently has the quantitative relationship between conformational population and physical properties been fully appreciated. This section, however, does not give extensive references to the experimental determination of the polysaccharide shape and size in different experimental conditions, but rather it attempts to focus on the molecular reasons for these perturbations. A digression is also made to include the electrostatic charges in polyelectrolytic polysaccharides, because of their diffusion and use and because of interesting variations occurring in these systems, including the marine polysaccharides discussed in the following section. In particular, since alginate and chitosan will be used for the application given in Section 4, the relation between the structural features of these compounds and their use in gelation systems are focused on here.

Alginate is a polysaccharide of algal or bacterial origin. It is composed of units of D-mannuronic acid (M) and L-guluronic acid (G) which form homopolymeric block structures along the chains, namely M-blocks and G-blocks interspaced by alternate MG sequences [64]. The original work on elucidation of alginate binding properties goes back to the seventies, pointing out the important relation between the cooperative sequence of guluronan residues [65,66,67] and the egg-box model proposed by Rees and coworkers [68,69]. This model has been central for the phenomenological description of alginate gelation, although more recently both a refinement and an extension of the original model shed some more light on the structural features of the “dimeric forms” of guluronan sequences involved in the chelation with calcium ions and responsible for the gel strength [70,71,72]. A recent review on alginate as a “biomaterial” [73] describes the role of several features of comonomer fraction and sequence in the ion-binding properties of alginate towards divalent ions and includes the extended description of the thermodynamic aspects of “egg-box” model and of the mechanism of hydrogel formation [74,75,76]. The concluding remarks on the gelation features of alginate attribute to calcium ions the right size and charge to fit into the distorted egg-box structure, including a non-negligible effect of the secondary junctions due to the alternating sequences of MG-type [77]. 

Chitosan derives from the chitin (extracted from crustacean and insect exoskeleton or from some algae and fungi) and is essentially a polyglucosamine varying in the degree of acetylation (DA) and molecular weight. The presence of a variable number of free amino groups and substitutions is responsible for the tunable interaction with anionic and polyanionic systems [78]. Although less modeled, chitosan conformational properties are mainly ascribed to the two-fold cellulose-like helix [79]. These features largely explain the intrinsic conformational stiffness as measured by several hydrodynamic and scattering experiments, sometimes affected in the past by uncertainties due to the microgel formation for samples at high DA [80]. Recent experimental results converge toward assigning to random acetyl substitution the relevant key-factor in controlling the changes in the chain stiffness, with a significant flexibility achieved at moderate to medium DA (<50%). This result is at least in part due to the common “co-monomer” effect, but must contain other contributing factors, such as short-range nearest-neighbor interactions [28] and long range excluded volume effect [81]. More recently, enhancement of chain flexibility and solubility properties has been reached by controlled chemical-etching of the glucosidic ring with periodate oxidation [82], as well as by introducing several types of pendant groups at the amino functionality [83].

## 3. Encapsulation in Polysaccharide Hydrogels

### 3.1. Polysaccharide-Based Hydrogels for Technological Applications

In biomaterial science, hydrogels are defined as “three-dimensional, hydrophilic, polymeric networks capable of imbibing large amounts of water or biological fluids” [84]. Such networks can be chemically (covalently) or physically cross-linked (by reversible molecular entanglements, ionic and hydrophobic interactions, H-bonds, *etc*.). Due to their high water content and soft consistency, hydrogels are very similar to natural living tissues, with performances that overcome the other classes of biomaterials. Generally, hydrogels are classified as natural polymer hydrogels, synthetic polymer hydrogels and combination of the two types. Several polymeric materials are employed as biomaterials, each one with specific properties which influence the hydrogel design parameters [84,85,86,87]. Among the natural polymers, polysaccharides have a wide application due to their peculiarities: they are abundant and obtained from renewable sources (such as plants, algae, bacteria); they present a large variety of composition and properties, not easily reproducible by synthetic routes; their production is generally easier and cheaper than for synthetic polymers. Thus, the number of polysaccharides investigated for technological applications of hydrogels is extremely extensive [88]. Polysaccharides are also employed as derivatives obtained by chemical or physical modification, to tailor the final properties of interest; moreover, biotechnology can enable the *in vitro* production of high levels of polysaccharides from micro-organisms [89,90,91]. 

In bio-oriented applications, polysaccharide hydrogels are exploited as immobilization matrices and protective structures (as scaffolds and capsules) for sensible materials, such as living cells and active compounds. The application to relevant technological fields (pharmaceutical, food, and biomedical) of hydrogels based on two representative marine polysaccharides, alginate and chitosan, will be reviewed. 

### 3.2. Pharmaceutical Applications

Pharmaceutical applications of hydrogels are based on their capability to act as exceptional drug delivery vehicles. Drugs and bioactive compounds are incorporated into the matrices and can be released according to various release profiles depending on the hydrogel properties [84,92]. It is generally accepted that the drug release from the matrix follows two main mechanisms, that is diffusion of the protein through the pores of the polymer network and degradation of the polymer network [93]. In addition, water diffusion and swelling through the hydrogel are two of the major factors affecting drug release rate [94]. Thus, drug release from a hydrogel is governed by several parameters: pore volume fraction; pore sizes; extent of interconnections; size and physico-chemical nature of the drug molecule, and in general type and strength of interactions between drugs and polymeric chains [86]. When alginate and chitosan are used for the preparation of hydrogel matrices for drug release, the fine control of the polymer structure and molecular weight [95,96,97,98], and of the conditions employed for the formulations (such as type and concentration of gelling ions, polymer concentration, procedures, *etc*.) [99,100,101], enable tailoring the functional properties of the drug delivery system. At a molecular level, the alginate hydrogel structure is strictly related to the composition and sequence of the polymer, and thus to the origin of the sample. Chitosan properties are also determined by the acetylation degree. All these factors deeply influence the characteristics of the hydrogel, and thus the capability to entrap, protect and release the drug payload. 

Some peculiar properties of hydrogels are shown in physiological conditions: for example, they can swell depending on the external environment, behaving as physiologically-responsive hydrogels. Some hydrogels show significant changes in the swelling ratio, and thus in release capability, in relation to pH, ionic strength, temperature or electromagnetic radiation. Moreover, hydrogels are used as muco-adhesive drug carriers able to interact with the mucosal surface (for example in the gastrointestinal tract or the respiratory epithelium) prolonging their residence time at the site of delivery. The interaction between carriers and mucosal glycoproteins occurs primarily via hydrogen bonding, and materials containing a high density of carboxyl and hydroxyl groups are chosen for these applications [84,102,103]. Hydrogel-based systems have been exploited for protection and delivery of low-molecular weight drugs and macromolecular payloads [104,105], such as peptide and protein drugs (insulin, melatonin, heparin, haemoglobin, parathyroid hormone, calcitonin, *etc*.) [106,107,108], nucleic acids (DNA, siRNA) [109,110] and antigens (from pathogens responsible of influenza, pertussis, diphtheria, tetanus, *etc*.) [107,111,112,113]. In addition to antigens, also vaccines have been encapsulated (attenuated or inactivated pathogens) in both medical and veterinary fields, combining the cell immobilization to a drug delivery system. Such technology is gaining great attention, due to the possibility to protect the vaccine during the administration (also for the oral route) and hopefully to control the targeting and delivery rate [114,115]. A detailed example of the application of biopolymer encapsulation of fish vaccinations will be presented in Section 4.2.

### 3.3. Other Applications

The oldest application of polysaccharide hydrogels is in food technology: in foodstuff, they are naturally responsible of the texture, so they have been used as thickening or gelling agents (for example in juices and chocolate). Nowadays, the use of microencapsulation with biopolymers has gained great importance in the development of food enriched with bioactive components (lipids, vitamins, peptides, fatty acids, antioxidants, minerals and also living cells, such as probiotics) and nutraceuticals (ingredients with potential health benefits [116,117,118]), for the protection of the compounds against inactivation or degradation, and also for their controlled delivery. The addition of such substances to food matrices is pursued to produce physiological benefits or reduce the risk of specific diseases. This implies a huge challenge, in common with pharmaceutical applications, since only a reduced amount of molecules remains available after oral administration, due to several factors, such as low permeability or solubility within the gut, and instability in the gastro-intestinal tract (due to pH, enzymes, presence of other nutrients) [117], or under food processing conditions (temperature, oxygen, light). Microencapsulation is used to stabilize reactive, sensitive, or volatile ingredients, and can be considered the source of totally new ingredients with exceptional properties, thanks to the tailored controlled-release properties: targeted and controlled release enhances the effectiveness of food additives, ensuring the optimal dosage, and (not marginally at all) improves the cost effectiveness [119]. Many encapsulation procedures have also been developed in the food industry: each technology suits different systems in order to meet the physico-chemical and molecular requirements of a specific bioactive component. 

A lot of GRAS biopolymers are compatible with many food components and are already used in the food industry (such as alginate, pectins, dextran, starch, cellulose and derivatives) and can combine a proper protection of the product and a targeted delivery. Often these polymers are modified to optimize the compatibility with the food matrices [118,120]. In principle, no real distinction exists between procedures used in food and in pharmaceutical technology, but of the entrapped molecules. 

In the biomedical technology (tissue engineering and regenerative medicine) hydrogels are the most relevant materials, thanks to their peculiarities, such as biocompatibility, physical properties, flexibility in the synthesis, and variety of constituents [121]. Hydrogels are currently used in several applications: as scaffolds, as tissue barriers and bioadhesives, as drug depots, as delivery systems for bioactive agents (that enhance the natural reparative process), as matrices to encapsulate and deliver cells [121]. Thus tissue engineering represents a great potential for the regeneration of tissues and organs, as an alternative to the traditional approaches to tissue or organ failure, mainly based on the replacement of the tissue with a synthetic implant, on the transplantation of the organ, and also on extracorporeal treatments (passive membrane exchange, experimental biohybrid systems, *etc*.). Among several strategies, the most appealing is based on the combination of cells and polymer scaffolds [86]. The 3D-scaffolds are used as substrates for supporting and guiding tissue regeneration in several *in vitro* and *in vivo* systems which mimic the extracellular matrix (ECM), a structure made of protein and sugar-based macromolecules, that provides the physical and biological support for cell and tissue growth. The preparation of ECM-mimicking biomaterials with defined shape and complex porous architecture represents still a technical challenge [122]. Hydrogels designed as 3D-scaffolds should present pores with an ideal size to accommodate living cells; moreover, they can be designed to dissolve, releasing active molecules [85,86]. Nowadays a variety of tissues has been engineered, with systems at the stage of clinical trial or application, including artery, bladder, skin, cartilage, bone, ligament, and tendon [85,123].

The cell immobilization and encapsulation systems have a broad range of applications: one of the most studied and described is the transplantation of microencapsulated cells, proposed as a therapy for the treatment of a wide variety of diseases. This technology is based on the principle that foreign cells (xenogeneic or allogeneic) are physically segregated and protected from the host immune system by an artificial membrane [124,125,126]. Thus hydrogel microcapsules can provide immunoisolation, without interfering with the diffusion of oxygen, nutrients, and metabolic products [121]. Various cell types including primary cells, stem cells [127] or bioengineered cells have been investigated for the treatment of the different diseases [128]. Some relevant examples are the treatment of cardiovascular diseases, kidney and liver failure, and diabetes mellitus (by the microencapsulation of islets of Langerhans) [123,129,130]. 

### 3.4. Methods for Microencapsulation

As previously reported, the encapsulation in polymeric hydrogels is employed to protect a wide range of materials of biological interest, from small molecules to cells (of bacterial, yeast, plant and animal origin). As a general definition, microencapsulation is “the technology of packaging solid, liquid and gaseous materials in small capsules that release their contents at controlled rates over prolonged periods of time” [116]. A variety of methods of encapsulation has been developed, and the best choice is based on the final requirements, such as mean particle size, physical/chemical properties, payload application, desired release mechanisms, industrial manufacturing scale and the acceptable processing cost [123]. 

Among all the existing methods, here the attention will be primarily given to the microencapsulation by ionotropic gelation which is one of the most employed, by exploiting the capability of some marine polymers to form hydrogels in the presence of proper multivalent counterions (such as calcium ions for alginate [124] or triphosphate for chitosan [131]). The protection is based on the embedding effect of the polymeric matrix, which controls interactions between the internal and external parts. Although such versatile technology is widely used in the technological fields of food industry, pharmaceutics and biomedicine, the variety of procedures requires some systematization that necessarily includes the other methods. 

**Figure 5:**
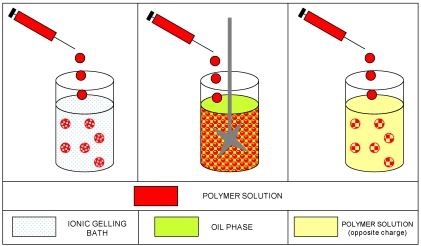
Schematic representation of the methods described for gel particle technology.

In the literature definition [93,116], gel particle technology mainly includes three different methods to prepare microspheres by ionotropic gelation (as schematically represented in Figure 5): (a) dropping the polyelectrolyte solution into a solution of small ions; (b) via a water in oil emulsification technique; and (c) complexation of oppositely charged polyelectrolytes by mixing, with additional coating procedures. These original classifications merged over the years into other approaches that use, indeed, several different techniques and procedures, often generating a confusion of terms.

**Figure 6:**
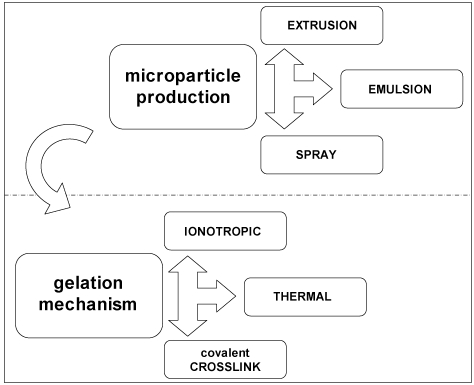
Graphical classification of the encapsulation procedures and techniques in polysaccharide hydrogels.

In the attempt to rationalize the several approaches into the frame outlined in the previous sections, a graphical classification of the procedures and techniques to encapsulate in polysaccharide hydrogels is proposed in Figure 6. The microparticle production is regarded as a preliminary step for the physical generation of spherical microdomains, independently of the final stage of preparation. Thereafter, whichever production method of microparticles is used, the use of an appropriate gelation mechanism is foreseen to stabilize the microdomains.

Thus, in the following part, the methods to obtain microscale droplets/particles and those to transform them in hydrogels will be separately described by reviewing some recent literature classifications [116,123,132,133]. The focus will be on the encapsulation in hydrogels of biological entities (biomolecules, drugs, *etc*.), taking into account that the literature nomenclature for the procedures could vary in different application fields [118]. 

### 3.5. Microparticle Production

The first step in the preparation of hydrogel microcapsules is to obtain micro-droplets which will then be gelled in different ways. Three procedures will be presented: extrusion, emulsion and spray-technologies.

The “extrusion” technique is widely exploited and, in the simplest case, can be performed by using a syringe with a needle. As a general principle, the polymer solution is extruded through an orifice and dripped into a hardening (gelling) bath. The size of the droplets determines the final dimensions of the gel particles, and can be controlled through several system parameters: the diameter of the orifice, the flow rate, the viscosity of the solution, the distance between the hardening solution and the orifice, the polymer concentration and the temperature [123]. The reduction of the droplet (and gel particle size) is a fundamental technical problem, which has been handled by developing several devices based on different physical mechanisms. The most used are the coaxial laminar air-flow, the air atomization, the electrostatic potential, the vibrating nozzle, the jet cutting and the spinning disk atomization [134,135,136]. The resulting particle size is not the only factor to be considered since other aspects are relevant, such as reproducibility, size tunability and distribution, time and yield of production. 

Emulsification is the process of dispersing a liquid in a second liquid that is immiscible [118]. The resulting emulsion can be defined basically as a system of two phases (dispersed and dispersing) in which droplets of one liquid are dispersed in another: when the emulsion is made of oil droplets in an aqueous phase it is called “oil-in-water” (O/W), while the opposite system is called “water-in-oil” (W/O). The stability of an emulsion can be regulated by the addition of a surfactant. By working on the composition of the two phases, the type of polymer and surfactant, this technique has been employed also for the production of nanometric droplets and particles. Advances in this technology have been obtained also by the application of microfluidic principles [137,138]. 

Among the spray-technologies, spray-drying is based on the formation of dried particulate starting from a fluid material (a solution, an emulsion or a suspension), by the atomization in heated gas (air or nitrogen), and the fast removal of the solvent (water). The powder particles are then separated from the drying air. This continuous process is influenced by the temperatures of the system (feed, air inlet and air outlet temperatures), the viscosity and concentration of the feed solution, the morphology of the polymer. Hydrogel particles can thereafter be produced by re-hydrating the powders under gelling conditions. Other methods, such as spray-cooling and spray-chilling, are based on a different principle and in the absence of solvent: the dispersion of a bioactive product in a polymeric matrix is cooled (or chilled) to allow solidification and immobilization [118,123,139]. Finally, the spray-coating technology is often reported in association with gel particle production. It is an efficient coating technique, based on devices such as the fluidized bed, to be used with virtually any polymer. The core particles are suspended by an air flow and then coated with the polymer solution sprayed from different directions (top, bottom and using a tangential spray). This technique can be used on hydrogel microcapsules after the gelation step [116,123]. 

### 3.6. Gelation Methods

The step that follows the preparation of micro-droplets of the desired dimension is the gelation, which can occur in different ways through physical and chemical mechanisms.

Physical gelation is mainly done by ionotropic and thermal gelation. The ionotropic gelation is in turn carried out by two main techniques, diffusion and internal setting. The first one is based on the introduction (mainly a dripping) of the polymer solution into an ionic solution: gelation occurs when the ions diffuse into the polymer solution droplets. The second is based on the addition of gelling ions in inactive form (such as calcium carbonate for calcium alginate gels). The gelation is triggered by changes in some system properties, generally pH changes [123]. The ionotropic gelation is suitable for marine biopolymers such as alginate, chitosan, carrageenan and also for materials of different origin (for example pectins). Microencapsulation in alginate hydrogels is probably the most exploited [140,141,142,143].

Also polyelectrolyte complexation (PEC), between polymers of opposite charges, is associated to the hydrogel formation, either as method to form the gel structure, or as technique to improve the quality (mechanical strength and permeability barrier) of hydrogel beads already prepared. In the latter case, the complexation can be concurrent with the ion-based gelation, or subsequent (coating step). PEC formation between chitosan and alginate (or other biopolymers) has been exploited for a long time, mainly for pharmaceutical applications for the production of micro- and nano-particles based systems [144,145,146,147,148].

Thermal gelation is applied to agarose, as marine polysaccharide, but also to gelatin, maltodextrin and several synthetic polymers, such as polymethylmethacrylate, polystyrene, poly(*N*-isopropyl-acrylamide), *etc*. Some hints for understanding the theory of this phenomenon have been given in Section 1. Concerning cell encapsulation, the agarose-based hydrogels have been widely studied for several biological applications [149,150]. 

Chemical gelation is based on the covalent cross-linking of the polymer chains, which results in the formation of the 3D-matrix. Several examples have been reported in literature, the oldest being the reticulation of dextrans to prepare gel beads for size-exclusion chromatography. The formation of chitosan microcapsules was initially based on the glutaraldehyde cross-linking. However, its use is avoided in cell and human applications because of the toxicity of such a reactant. Covalent cross-link can be obtained also by enzymatic routes, among which the safe cross-linker genipine has been employed for the preparation of hydrogels and beads of chitosan [88]. There is also a long list of covalent cross-linking reactions induced by the exposure to UV or visible light (photo-crosslinked hydrogels) [151]. 

In addition, several strategies of combinations of physical and chemical hydrogel formation techniques have been proposed, leading to “in tandem” mechanisms, in order to tailor and enhance the properties of the microcapsules produced [152,153,154]. 

It is also mandatory to mention the application of the natural biopolymers at the nanoscale level. Biopolymeric nanocapsules, nanogels and nanoparticles have been developed since the early nineties (in parallel to the liposome and the other lipid-based nanosystems) with the logical application to the encapsulation of biological entities of smaller dimensions (mainly drugs, therapeutic proteins, nucleic acids) [155,156,157,158]. Drug delivery systems based on polysaccharide nanoparticles present several advantages: the capability to penetrate cells and tissues; the possibility to improve the bioavailability of drugs, reducing toxic side effects; the ability to control release properties due to the biodegradability and the stimuli (pH, ion and temperature), and sensibility of materials [146]. The rationalization of the assembly mechanisms and the capability to tailor the properties (size, charge, and loading capability) to desirable levels are essential goals to advance biodegradable polysaccharidic nanoparticles as efficient drug delivery vehicles. Nowadays the results obtained by the researchers in this field have generated a huge number of patents [159,160] and remarkable scientific production [138,161,162,163,164,165].

## 4. Microencapsulation for Fish Vaccination in Aquaculture: A Case Study

### 4.1. Fish Vaccination in Aquaculture

Aquaculture represents a fast growing sector of the food industry, providing one half of the fish consumed by humans [166]. Over the past three decades, aquaculture has expanded, intensified, and diversified. However, the intensive production of fish farming results in stress for fish and health problems, such as increased vulnerability to disease outbreaks. This situation influences dramatically the economic and socio-economic development of many countries [166,167].

The prevention of diseases, by using optimal husbandry practice and biological control methods (such as vaccination and the use of immunostimulants) is mandatory, due to the concerns regarding the environmental pollution associated with chemical treatments, the emergence of multiple resistance to antibiotics, and the resulting consumer unsafe products [168]. At an international level, the urgent requirement of pro-active and reactive programs has been expressed, to solve health questions in order to sustain the growth of aquatic animal food production [166]. 

Currently, the applied research is oriented towards the development of new preventive strategies based on fish vaccinations. This practice has a great relevance in large-scale commercial fish farming and, for example, has been responsible for successful salmon cultivation. In general, commercial vaccines are based on inactivated bacterial pathogens, but at the moment few viral and no parasite vaccines are commercially available [169]. In this context, oral vaccinations are appealing since the first contact with pathogens occurs through the mucosal surfaces of the gut at the level of the gut-associated lymphoid tissue (GALT). Fish intestine indeed does not present Peyer’s patches and antigen-transporting M cells: however lymphoid cells and macrophages are present between the epithelial cells and in the lamina propria, and enterocytes show an antigen-transporting capacity [170]. Compared to intra-peritoneal injection, oral administration of vaccines is simple, cost-effective, stress free and easy to administer to large numbers of fish at one time, thus being suitable for mass fish immunization. Oral vaccines are generally produced in two ways: by top coating the feed with the antigen or by preparing a mix with the feed during the production. Oral vaccination at the moment presents also relevant disadvantages, such as the generally high amount of antigen required to provoke an immune response; the lack of an adequate duration of the immunoprotection; the damages from digestive hydrolysis; the necessity to enhance the uptake by the hind gut, in order to induce an effective protective immune response; the physiological and anatomical differences among the species (gastric or agastric fish) [115,167]. These limitations led researchers to develop methods of protecting the vaccine, and thus new fish vaccines and advanced technology have been implemented. For a systematic overview on the most diffuse fish diseases, the vaccines already available, the species that are treated and the developments in the delivery strategies, the reader is addressed to some relevant review publications [115,169,171].

### 4.2. Case Study

Part of the work here summarized as a case study has been set up in the frame of a Research Project [172] in which a network of scientific institutions and fish farming plants shared their competences to develop an oral vaccination protocol for lactococcosis in trout, exploiting the administration of the inactivated bacteria microencapsulated in biodegradable biopolymeric matrices. Lactococcosis by *Lactococcus garvieae*, is one of the most relevant fish diseases in intensive aquaculture, causing substantial economic losses to rainbow trout (*Oncorhynchus mykiss*) farming [173]. Microencapsulation has been employed since the late nineties as a way to protect the vaccine to be delivered in aquaculture. Three polymers (alginate, chitosan and PLGA) have been mainly used to encapsulate various pathogens (such as *Vibrio anguillarum*, *Lactococcus garvieae*, *Aeromonas hydrophila*) or nucleic acids (for example DNA plasmids containing genes coding for antigens), in order to treat several fish species (carp, Japanese flounder, trout, Nile tilapia, *etc*.) [115]. The results so far obtained for the specific case of alginate encapsulation of the lactococcosis pathogen show that the system is still not optimal: at the moment it resulted mainly in a successful boost, in association with the traditional injection [173,174]. 

The set up of a microencapsulation system for large scale production of vaccine loaded microcarriers is technologically very challenging. The system developed in our study [175] is an upgrade of a previous one, produced on a laboratory scale, that has been successfully tested in preliminary trials, both on fish (sea bass) and mammal (mice) models [176,177]. It is based on a mixture of biopolymers that have been chosen among other candidate biomaterials. In particular, alginate, chitosan and a cellulose derivative (hydroxypropyl methyl cellulose, HPMC) have been used in a procedure, based on an ionotropic gelation combined with a polyelectrolyte complexation. Lysozyme has been also added as adjuvant and co-encapsulated with the vaccine, due to its immunomodulating properties [178]. The system has been fully characterized and has been tested in two seasonal trials; the evaluation of the results is not the object of this paper.

The preparation of the microcapsules on a large scale [179] has been paralleled by the set-up of a model system (polymer beads), obtained at bench scale by a simple extrusion technique [180]. The feeding solutions and the beads have been characterized for properties [181,182] derived from the combination of different materials (alginate, chitosan, HPMC and lysozyme). The compositional effect on the encapsulation process and formulation is detected also from bead shape, transparency and morphology. The rationale for the employment of a simple model system comes up from the necessity to collect information regarding the role of each component and their mutual interactions within the system. Moreover, the effects of the polymer nature (such as type and origin of alginate samples, or chitosan characteristics) on the resulting hydrogels have been studied and optimized. 

The composite biopolymeric beads can be used as a general model of drug delivery system, and thus have been tested in a variety of “environmental” conditions (pH, temperature, ionic strength, *etc*.) which correspond to different physiological conditions (mimicking, for example, gastric or intestinal release, stomach-less or stomach-containing fish, *etc*.), thus enabling the investigation of both human and veterinary applications. 

The application presented here, typically follows the understanding of the concepts exposed in the previous sections of this review; in that sense it is not only a case study, but also a real “proof of concept” in the field. As reported in Section 3.2, drug release is influenced by several parameters, which are dependent on the matrix structure (pore size, pore volume fractions, extent of the interconnections, *etc*.) and from the surrounding environment (such as pH, ionic strength, temperature, chemical composition, *etc*.). For this reason, the effect of the composition of the buffer medium on the release of the encapsulated protein has been also evaluated. Release studies have been carried out in buffer medium to guarantee the maintenance of the pH (phosphate buffer and Tris buffer with the addition of NaCl at pH = 7.4). 

**Figure 7:**
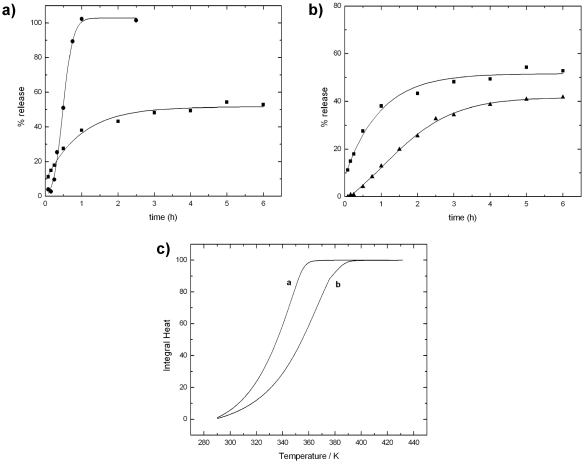
(**a**) Comparison of lysozyme release from alginate beads in phosphate buffer (●) and in Tris/NaCl (■) at pH = 7.4; (**b**) Comparison of lysozyme release in Tris/NaCl from alginate beads (■) and from alginate/chitosan beads (▲); (**c**) Experimental integral heat of water evaporation from alginate beads (curve a); and from alginate/chitosan beads (curve b). Polymer samples: alginate (F_G_ = 0.4; Mv = 86 kDa); chitosan (Mv = 492 kDa; DA = 11%). Other experimental details in refs [182,183].

Figure 7a reports the release profiles of lysozyme from alginate beads in phosphate buffer and in Tris/NaCl. In phosphate buffer, release occurs very rapidly and it is completed during the first hour. The rapid release kinetics is not due to the pH value, but rather to a fast erosion of the alginate matrix in the presence of phosphate ions, which subtract calcium ions from the guluronate sites of the gel (see egg-box model illustrated in Section 2.5). In fact, a different behavior is observed in Tris/NaCl buffer. The use of Tris prevents the matrix from erosion and the release of lysozyme is partially hampered. 

The alginate gel matrix can be reinforced by employing other polysaccharides able to interact with alginate either with electrostatic and/or dipolar interactions. This structurization is an effective hampering factor to the rapid release of proteins. The two polymers here exploited have been already largely used in the past, one is chitosan [97,184], and the other is the cellulose derivative HPMC. Both polymers have been employed in order to modify the barrier properties of the matrix. Cellulose derivatives, like HPMC, are also commonly used as a polymer generating swellable matrix, since in solid tablets it undergoes a glassy-rubbery transition as water penetrates [185,186]. Figure 7b reports the release profiles of lysozyme in Tris/NaCl from the alginate beads and the chitosan-reinforced alginate beads. In Tris buffer, the main process occurring is diffusion. The effect of the addition of chitosan is clear from the release profile, which is lower than that of the sole alginate. 

The idea that the addition of other polysaccharide to the basic alginate matrix produces a more intricate matrix has been already modeled in the literature and it is referred as “obstruction effect” [187]. In a previous study [183], a calorimetric approach has been proposed to measure this effect. Indeed, differential scanning calorimetry has proven to be a powerful tool to study the barrier properties of the different systems. In particular, water evaporation rate from beads of different compositions, obtained by adding HPMC and/or chitosan, has been investigated to evaluate the effect of additional carbohydrate polymers on the alginate matrix [182]. The rationale of this approach is that the study of water evaporation can be helpful in understanding the structural arrangement of the polymer chains in the beads. It is claimed that the shift of the thermograms, arising from the addition of other components, reflects the “obstructive” character of the polymer network in the bead. Therefore, it is expected that a delay of water evaporation is paralleled by a decrease in protein release as it is shown in Figure 7c which reports the experimental integral heat of water evaporation from alginate and alginate/chitosan gel beads.

The addition of chitosan produces a delay of water evaporation, thus confirming the effect of chitosan, as demonstrated also by release data. The results of several experiments carried out to compare water evaporation and protein release from gel beads with different compositions are very helpful in predicting the release properties of new formulations by a rapid analysis. Besides the above reported tests and the positive response of this system to the “*in vitro*” analyses, trials in the field have shown remarkable but, as yet, not fully analyzed results; this is also due to some unavoidable variability in the trial conditions when compared to a laboratory set-up. 

The superior properties of the mixed alginate/chitosan with a proper addition of a little amount of an extra-marine polysaccharide (HPMC) is the reason for stating that the systems made with polymers from the sea, contribute to solve a problem in the sea. 

## 5. Conclusions

The overview provided in this paper has been mainly devoted to the elucidation at the molecular level of the main polysaccharide properties and of the processes on which the encapsulation in polysaccharides is based. This methodological approach to the comprehension of the physico-chemical properties of the systems being developed enables their efficient exploitation and provides a solution to technical and experimental problems, often encountered, but not always reported in literature. 

The superior properties of the polysaccharide-based systems described here and reviewed provide the reasons for stating that systems made with biopolymers from the sea may contribute to solve a problem in the sea, although limited to the aquaculture environment. 
